# Timing of use of cod liver oil, a vitamin D source, and multiple sclerosis risk: The EnvIMS study

**DOI:** 10.1177/1352458515578770

**Published:** 2015-12

**Authors:** Marianna Cortese, Trond Riise, Kjetil Bjørnevik, Trygve Holmøy, Margitta T Kampman, Sandra Magalhaes, Maura Pugliatti, Christina Wolfson, Kjell-Morten Myhr

**Affiliations:** The KG Jebsen Centre for MS-Research, Department of Clinical Medicine, University of Bergen, Norway/Department of Global Public Health and Primary Care, University of Bergen, Norway/Department of Nutrition, Harvard T.H. Chan School of Public Health, USA; Department of Global Public Health and Primary Care, University of Bergen, Norway/The Norwegian Multiple Sclerosis Competence Center, Department of Neurology, Haukeland University Hospital, Norway; Department of Global Public Health and Primary Care, University of Bergen, Norway/Department of Nutrition, Harvard T.H. Chan School of Public Health, USA/The Norwegian Multiple Sclerosis Competence Center, Department of Neurology, Haukeland University Hospital, Norway; Institute of Clinical Medicine, University of Oslo, Norway/Department of Neurology, Akershus University Hospital, Norway; Department of Neurology, University Hospital of North Norway, Norway; Department of Epidemiology and Biostatistics and Occupational Health, McGill University, Canada; Department of Global Public Health and Primary Care, University of Bergen, Norway/Department of Clinical and Experimental Medicine, University of Sassari, Italy/Division of Medicine, McGill University, Canada; Department of Epidemiology and Biostatistics and Occupational Health, McGill University, Canada/Research Institute of the McGill University Health Centre, Canada; The KG Jebsen Centre for MS-Research, Department of Clinical Medicine, University of Bergen, Norway/The Norwegian Multiple Sclerosis Competence Center, Department of Neurology, Haukeland University Hospital, Norway

**Keywords:** Multiple sclerosis, vitamin D, timing, environmental risk factors, susceptibility, age, supplementation, cod liver oil

## Abstract

**Background:**

Low vitamin D levels have been associated with an increased risk of multiple sclerosis (MS), although it remains unknown whether this relationship varies by age.

**Objective:**

The objective of this paper is to investigate the association between vitamin D_3_ supplementation through cod liver oil at different postnatal ages and MS risk.

**Methods:**

In the Norwegian component of the multinational case-control study Environmental Factors In Multiple Sclerosis (EnvIMS), a total of 953 MS patients with maximum disease duration of 10 years and 1717 controls reported their cod liver oil use from childhood to adulthood.

**Results:**

Self-reported supplement use at ages 13–18 was associated with a reduced risk of MS (OR 0.67, 95% CI 0.52–0.86), whereas supplementation during childhood was not found to alter MS risk (OR 1.01, 95% CI 0.81–1.26), each compared to non-use during the respective period. An inverse association was found between MS risk and the dose of cod liver oil during adolescence, suggesting a dose-response relationship (*p* trend = 0.001) with the strongest effect for an estimated vitamin D_3_ intake of 600–800 IU/d (OR 0.46, 95% CI 0.31–0.70).

**Conclusions:**

These findings not only support the hypothesis relating to low vitamin D as a risk factor for MS, but further point to adolescence as an important susceptibility period for adult-onset MS.

## Introduction

A low vitamin D level is one of the factors most consistently associated with multiple sclerosis (MS).^1^ Yet, it is not well understood at which age an adequate exposure might be especially important and an intervention optimally timed to modify MS risk.^2^

Observational studies investigating the timing of exposure reached different conclusions regarding the possibly most susceptible postnatal period: childhood,^3^ adolescence,^4^ childhood and adolescence,^5^ adulthood.^6^ Migration and space-time cluster studies also pointed to different postnatal susceptibility periods,^7–10^ though these findings could also reflect the effect of other environmental risk factors.^2^ Furthermore, lower vitamin D exposure and serum levels during the prenatal phase have been associated with increased risk of MS later in life and could mark an independent susceptibility period.^11–13^

Serum vitamin D levels are influenced by sun exposure and diet.^1^ Cod liver oil is an important dietary vitamin D source in high-latitude countries like Norway where there is no sun-induced vitamin D production during the winter.^14^ Norwegian Health Authorities have recommended 5 ml of cod liver oil daily (400 IU of vitamin D) for more than 60 years to prevent diseases like rickets, formerly more prevalent in areas with little access to vitamin D-rich fatty fish.^15^ A survey from 1997 estimated that about 35% of the Norwegian population and 50% of those aged 60–79 were still using the supplement on a daily basis.^16^

As the literature is inconsistent in delimiting a critical window in which vitamin D might act, we investigated the association between the postnatal timing of cod liver oil supplementation, an important oral vitamin D source in Norway, and the risk of developing MS.

## Methods

### The EnvIMS study

The multi-national multicenter case-control study of Environmental Factors In Multiple Sclerosis (EnvIMS) was launched to investigate environmental risk factors for MS and examine possible differences between distinct populations. A self-administered postal questionnaire (EnvIMS-Q) was designed including detailed questions about age-specific past exposures such as sun habits, diet, supplement use, past medical history, lifestyle, occupational and hormonal factors.^17^ The questionnaire has been assessed for acceptability, feasibility and reliability.^17^ The complete study design and methodology has been published elsewhere.^17^

The Norwegian component of the EnvIMS study was approved by the Regional Ethical Committee for Medical and Health Research for Western Norway (n. 11, 18.12.2008). All eligible study participants received an invitation to participate in the form of an information letter explaining the study objectives, relevance and instructions for participation. Completion and return of the questionnaire implied participants’ informed consent.

### Study population

These analyses used the Norwegian EnvIMS data collected in 2008. The cases, diagnosed according to the McDonald criteria,^18^ were recruited from the Norwegian MS-registry and Biobank.^19^ Only patients with disease duration shorter than 10 years were eligible for participation. Of the 1368 invited cases, 953 (69.7%) returned the questionnaire. The response rate was 72% among women as compared to 64.6% in men.

Controls, frequency-matched on age and sex, were randomly selected in a 4:1-ratio from the population-based National Registry in Norway (Folkeregisteret). Of the 4728 invited controls, 1717 (36.3%) participated. The response rate in women was again higher than in men (39.4% vs. 29.4%).

### Exposure, outcome and covariates

Cod liver oil is an important source of vitamin D in the Norwegian population.^16^ The recommended one teaspoon (two capsules) daily of cod liver oil (5 ml) of the most commonly used Norwegian brand (Möller’s, Axellus AS, Oslo) contains 10 μg (400 IU) of vitamin D_3_, 250 μg of vitamin A, 10 mg of vitamin E and 1.2 g of the omega-3 fatty acids EPA and DHA. A Norwegian survey estimated that the majority of consumers use one tablespoon (15 ml) as a serving size.^20^

Given the importance of cod liver oil as a source of vitamin D in Norway, the use of this supplement was explored in several questions in the EnvIMS-Q. Participants were asked to report whether they had used cod liver oil or capsules “never” or at ages “0–6,” “7–12,” “13–15,” “16–18,” “19–24” and “25–30.” The age-scale was adapted to the Norwegian school system. Additionally, the frequency of supplement use at ages 13–19 during the winter and the rest of the year was explored using two separate variables on a six-point scale including “never/seldom,” “1–3 times/month,” “1 time/week,” “2–3 times/week,” “4–6 times/week” and “7+ times/week.” Another question classified the usual quantity of cod liver oil consumed at each serving during the same period into “no use,” “half a teaspoon,” “one teaspoon,” “half a tablespoon” and “one tablespoon or more.” The special interest of the investigators in this period was based on previous findings indicating that adolescence might be especially important for MS risk modification.^21^

Further, data on important covariates were retrieved from the questionnaire. The level of sun exposure was estimated by summer outdoor activity at ages “0–6,” “7–12,” “13–15,” “16–18,” “19–24,” “25–30” and “in recent years,” and quantified as “not that often,” “reasonably often,” “quite often” and “virtually all the time.” The frequency of consumption of vitamin D-rich fatty fish at main meals at ages 13–19 was explored on a six-point scale with “never,” “1 time/month,” “2–3 times/month,” “1 time/week,” “2 times/week” and “3 and more times/week” for a) “herring,” b) “mackerel,” c) “halibut, flounder” and d) “salmon, trout,” respectively. From the information elicited about infectious mononucleosis, past occurrence of the disease was used as covariate (“yes,” “no” or “I don’t remember”). Participants also reported their body shape at five-year age intervals based on a nine-point scale derived from body sketches,^22^ a figure rating scale, which has been shown to reflect well individuals’ body mass index (BMI).^23,24^ Information on the smoking habits (smoking onset never or after versus before MS onset) and the participants’ level of education (elementary, middle, high school or university) was also included in the analyses.

Finally, participants reported whether they had a family history of MS (affected parent, sibling or child) and whether they had asked their parents or another person for help in recalling information.

### Statistical analyses

Statistical analyses were performed using STATA 13.1 (StataCorp, College Station, TX, USA). The associations between exposure and outcome were estimated through logistic regression and reported as odds ratios (OR) with 95% confidence intervals (CIs). All estimates were adjusted for sex and year of birth (six-year categories to create balanced subgroups).

According to the distribution of age at disease onset in the cases, an index age with corresponding distribution was assigned to controls taking into account age at time of study. Participants were considered exposed only if the exposure of interest occurred before the index postnatal age or age at disease onset. Participants exposed only after this period were considered unexposed.

Based on reported cod liver oil use at different ages, three variables were created: 1) cod liver oil use during childhood (ages 0–12) regardless of use during the other periods compared to no use during childhood, 2) cod liver oil use during adolescence (ages 13–18) regardless of use during the other periods compared to no use during adolescence and 3) cod liver oil use during adulthood (ages 19–30) regardless of use during the other periods compared to no use during adulthood. The effect of these variables was estimated in i) separate models adjusted for age and sex, ii) simultaneously in the same model and finally iii) also adjusted for different covariates.

Additionally, MS risk was compared for cod liver oil use continuously from birth up to different ages to investigate whether the duration of exposure was important. Information about frequency and quantity of supplement use at ages 13–19 was analyzed both as a categorical and a continuous variable using those who reported never having taken cod liver oil or capsules during a certain season as reference.

Consumption frequency of fatty fish during adolescence was examined by assigning scores between 0 and 5 to each participant to account for how frequently on the six-point scale “herring,” “mackerel,” “halibut, flounder” and “salmon, trout,” respectively, were consumed. These scores were added up to an overall score ranging from 0 to 20 reflecting the fatty-fish consumption. To facilitate analyses of the overall score, a quintile-inspired five-point scale from 1 to 5 was created grouping overall scores of 0, 1–2, 3–4, 5–6, ⩾7 and analyzed as a continuous variable.

Further, reported sun exposure during the summer at ages 0–12, 13–18 and 19–30, history of infectious mononucleosis, smoking prior to MS onset, body size at age 15, frequency of consumption of oily fish at ages 13–19 and years of education were added to the model to adjust for possible confounding. In a second step interaction terms were created to test for effect modification by sex and age at disease onset.

## Results

Mean study age and sex distribution were similar in both groups (Table 1). Cases were significantly more likely than controls to have smoked before MS onset, experienced infectious mononucleosis, reported a lower educational level, a large body size at age 15 and infrequent summer sun exposure during adolescence.

**Table 1.** Selected characteristics of the Norwegian participants in EnvIMS^a,b^.

	Cases (*n* = 953)	Controls (*n* = 1717)
Age at study, mean (SD)	44.8 (10.5)	46.0 (10.8)
Male, *n* (%)	286 (30.0)	461 (26.9)
Age at disease onset, mean (SD)	37.6 (10.2)^g^	n.a.
Disease duration, mean (SD)	7.2 (2.7)^g^	n.a.
Smoking before MS onset^c^, *n* (%)	545 (58.9)	853 (50.7)
Infectious mononucleosis, *n* (%)		
“Yes”	160 (17.3)	155 (9.3)
“No”	729 (78.7)	1486 (88.8)
“I don’t remember”	37 (4.0)	33 (2.0)
Educational level, *n* (%)		
High school or lower	538 (57.1)	802 (47.3)
University career	402 (42.7)	890 (52.5)
Body size at age 15, *n* (%)^d^		
Normal (silhouette 1–4)^e^	794 (87.0)	1486 (89.5)
Large (silhouette 5–9)^f^	119 (13.0)	174 (10.5)
Summer sun exposure, *n* (%)		
Lower vs. higher at age 13–15	302 (32.7)/623 (67.4)	456 (27.4)/1210 (72.6)
Lower vs. higher at age 16–18	487 (52.4)/443 (47.6)	779 (46.8)/885 (53.2)

EnvIMS: Environmental Factors In Multiple Sclerosis; SD: standard deviation; *n*: count; n.a.: not applicable.

a Missing data for covariates ranging from 0% to 3.6%.

b Cases asked significantly more often for help than controls to recall information when filling out the questionnaire (mother: 34.5 vs. 17.2%, father: 6.9 vs. 2.5%, other person: 8.3% vs. 4.2%).

c Only 0.9 % in this group smoked less than three years.

d Based on Stunkard’s figure rating scale.

e Body mass index (BMI) ranges from 18.9 to 23.5 kg/m^2^.

f BMI ranges from 26.1 to 43.3 kg/m^2^ including overweight and obesity.

g Based on data from the Norwegian MS-registry and Biobank.

### Timing of cod liver oil use

Supplementation habits did not vary with sex, but with age at the time of study. Supplementation during childhood, adolescence and adulthood was more common among participants born before 1962.

Cod liver oil use was reported by 54.4% of cases and 55.9% of controls during at least one of the age ranges of interest between birth and age 30. Information on the age-specific supplementation was missing for 11.6% of participants. The association between the risk of developing MS and cod liver oil use varied considerably depending on the timing of the supplementation (Table 2). A marked inverse association was observed for intake at ages 13–18 after adjusting for age, sex and supplementation during the other periods. Neither cod liver oil use during childhood nor adult life was associated with reduced disease risk compared to non-use during those periods.

**Table 2.** Association between cod liver oil use at different ages and the risk of MS.

	Use compared to no use of cod liver oil during		
	Childhood (0–12 y)^a^	Adolescence (13–18 y)^a^	Adulthood (19–30 y)^a^
Cases *n* (%)	301 (31.8)	196 (20.6)	165 (17.4)
Controls *n* (%)	631 (36.9)	473 (27.6)	309 (18.0)
	*OR (95% CI)^b^*	*OR (95% CI)^b^*	*OR (95% CI)^b^*
Model 1^c^	0.82 (0.69–0.97)^f^	0.70 (0.58–0.85)^g^	1.00 (0.81–1.24)
Model 2^d^	1.01 (0.81–1.26)	0.67 (0.52–0.86)^h^	1.17 (0.93–1.47)
Model 3^e^	1.01 (0.79–1.29)	0.72 (0.55–0.96)^f^	1.18 (0.92–1.51)

MS: multiple sclerosis; y: years; *n*: count; OR: odds ratio; CI: confidence interval.

a Continuous or periodical use regardless of prior and subsequent use.

b OR of MS for age at disease onset after the exposure period of interest by cod liver oil use during specific age periods compared to no use during the same period.

c Model 1: Separate model for each age period. Adjusted for age and sex.

d Model 2: All three age periods included in the same model. Adjusted for age and sex.

e Model 3: All three age periods included in the same model. Adjusted for age, sex, smoking before disease onset, history of infectious mononucleosis, sun exposure, body shape at age 15, education, and consumption of fatty fish.

f *P* value < 0.05.

g *P* value < 0.0001.

h *P* value < 0.005.

The association between cod liver oil supplementation during adolescence and MS risk was not meaningfully altered after adjusting for sun exposure, infectious mononucleosis, smoking, body size, oily fish consumption and education. No significant differences were seen in the effect between men and women and according to age at disease onset (data not shown).

We observed that 404 cases (42.4%) and 368 controls (21.4%) asked for help in completing the questionnaire. Of these, 34.1% vs. 42.2% used cod liver oil during childhood, 19.8% vs. 30.2% during adolescence and 15.2% vs. 19.4% during adulthood, comparing cases and controls, respectively. When restricting the analysis of the fully adjusted model 3 (Table 2) to those asking for help, the pattern of association remained similar. The respective OR (95% CI) were 0.99 (0.66–1.49) for childhood, 0.67 (0.42–1.06) for adolescence and 0.99 (0.64–1.54) for adulthood use.

**Figure 1.**
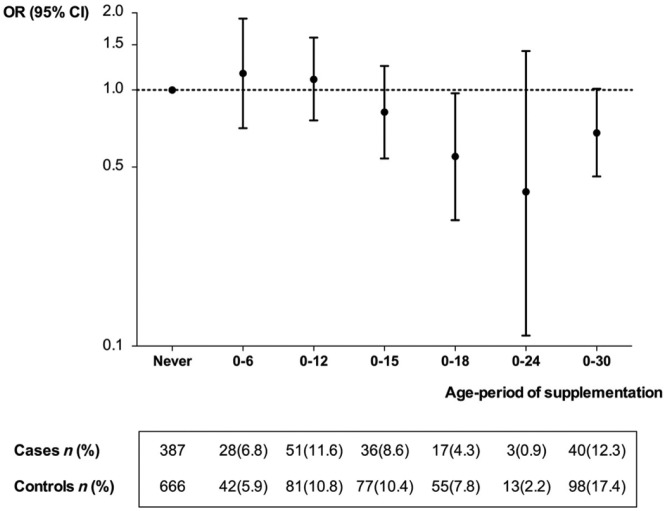
Association between cod liver oil use during increasingly longer age-periods and MS risk. MS: multiple sclerosis; OR: odds ratio; CI: confidence interval. n: count. OR of MS in groups with cod liver oil use continuously from birth up to a specific age compared to those who reported no use in the past and number of cases and controls.

Continuous supplementation from birth up to a certain age was increasingly more strongly associated with a reduced MS risk the longer the supplementation lasted, except for the longest period from birth to age 30 (Figure 1).

### Supplementation during adolescence

Supplementation during adolescence was further analyzed for seasonality, frequency and habitual serving size.

The habit of using (liquid) cod liver oil during adolescence was more common during the winter (33.7% and 43.6%) than the rest of the year (19.7% and 23.9%), both in cases and controls. Information was missing for 15.9% of participants for intake during winter and 29.3% during the rest of the year. The frequency of intake most often reported during adolescence in the winter, if any, was “7+ times a week” both for cases (41.8%) and controls (39.3%). The serving size most commonly reported was “1 tablespoon” for cases (58.7%) and controls (64.3%).

Table 3 shows the association between MS risk and increasing doses of vitamin D_3_ as estimated from the frequency and quantity of supplementation in the winter during adolescence, suggesting a dose-response relationship (*p* trend = 0.001) and the strongest protective effect for 600–800 IU/d. The estimates did not substantially change after adjusting for a selection of confounders (Table 3), nor after also adding family history of MS into the model.

**Table 3.** Association between average daily intake of vitamin D_3_ through monthly supplemented cod liver oil in the winter during adolescence (ages 13–19) and MS risk for age at disease onset >19.

*Cod liver oil(ts/month)*	*Vitamin D_3_(IU/day)*	Cases *n* (%)	Controls *n* (%)	OR^a^	95% CI
None^b^	–	525 (66.0)	784 (56.1)	1.00	–
1–15	⩽ 200	79 (9.9)	160 (11.5)	0.74	0.55–0.99^c^
16–30	201–400	55 (6.9)	125 (9.0)	0.68	0.48–0.95^c^
31–45	401–600	14 (1.8)	38 (2.7)	0.58	0.31–1.08
46–60	601–800	32 (4.0)	104 (7.4)	0.46	0.31–0.70^d^
>60	>800	90 (11.3)	186 (13.3)	0.77	0.58–1.02

MS: multiple sclerosis; *n*: count; ts: teaspoons; IU: international units; OR: odds ratio; CI: confidence interval.

a All estimates adjusted for age and sex; p-trend for association = 0.001, OR (95% CI) = 0.91 (0.87–0.96). Adjusting in addition for smoking before disease onset, history of infectious mononucleosis, sun exposure, body shape at age 15, education, and consumption of fatty fish: *p* trend = 0.025, OR (95% CI) = 0.94 (0.89–0.99). The effect estimate did not materially change when adjusting in addition for supplementation during adolescence in the summer.

b Reference group consists of those who reported neither an intake of cod liver oil liquid nor capsules.

c *P* value < 0.05.

d *P* value < 0.0001.

The association was significant for supplementation only during the winter. For the supplement use in other seasons we found no evidence of an association between doses of cod liver oil during adolescence and MS (OR 1.03, 95% CI 0.84–1.26, *p* trend = 0.773, adjusted for supplementation during adolescence in the winter and the covariates listed in the methods).

### Fish consumption

Consumption of fatty fish during adolescence was associated with a significant reduction in risk for later MS development (OR 0.92, 95% CI 0.86–0.99, *p* trend = 0.047, adjusted for all covariates mentioned in the methods). This OR was related to one step on the five-point scale and is equivalent to an OR of 0.72 comparing most frequent with no fish consumption. When further adjusting for cod liver oil use during adolescence, the result did not meaningfully change, but was no longer significant (OR 0.93, 95% CI 0.86–1.01, *p* trend = 0.085).

## Discussion

We found an inverse association between MS risk and cod liver oil supplementation during adolescence and a dose-response protective effect suggested for higher dosages of vitamin D consumed through cod liver oil. However, no association was observed for supplementation during childhood or adulthood. These findings suggest that adolescence might be an especially susceptible period for disease risk modification through dietary vitamin D and are in line with previous observational^4,21^ and experimental^25^ studies. Other environmental risk factors like infectious mononucleosis,^26^ high BMI^24^ and other lifestyle factors^27^ have also been suggested to act mainly during adolescence.

Previous studies in the same area found that sun exposure through outdoor activity during adolescence was associated with a decreased disease risk only when exposure occurred during the summer,^4,28^ while the association between MS and cod liver oil use during adolescence in the present study was only significant for intake during the winter when sun-induced vitamin D-production ceases. These seasonal differences suggest that the risk-modifying effect is vitamin D mediated.

A low vitamin D level is one of the risk factors most consistently associated with an increased risk of MS. A prospective study reported a decreased MS risk among adult nurses comparing supplemental intake of ⩾400 IU/d of vitamin D to no intake.^6^ However, it is unclear how vitamin D levels in adulthood correlate with those during adolescence.

Our findings indicate an especially sensitive period during adolescence for MS risk modification but probably not the only one.^21^ Higher doses of vitamin D may be needed during childhood and adulthood to reach the same degree of risk modification as during adolescence.^21,25^ Even though we did not find an association between overall cod liver oil use during childhood or adulthood and MS risk, we could not evaluate whether a protective effect was restricted to high-dose users at these ages, as this information was not collected.

A prospective study in the United States (US) did not find an association between total recalled dietary vitamin D intake during adolescence and MS risk.^29^ Intake of ⩾400 IU/d of vitamin D from multivitamins during adolescence showed, however, a nonsignificant reduced MS risk of an order of magnitude similar to the findings in our study under the rare-disease-assumption.^29^ Power might not have been optimal to yield significant results particularly since diet contributes only to a small extent to the vitamin D status compared to sun exposure in the studied area.^29^ In the area of the present study there is virtually no contribution of sun exposure during winter.

There are alternative explanations to our findings. Adolescence might be the most sensitive period in which vitamin D unfolds its observed immunomodulatory effects^30^ or is of importance in the terminal phase of brain development. Another explanation could be that vitamin D supplementation during different periods in life might not lead to comparable serum levels. No age-dependent difference was, however, experimentally found in how dietary vitamin amounts translate into serum levels.^25^

Our results might also be due to chance, but this is unlikely considering the strong association and conformity with previous findings. We adjusted the estimates for other known environmental risk factors, but we cannot exclude the possibility of residual confounding.

Alternatively, the inverse association between cod liver oil use during adolescence and MS risk might be due to a longer period of exposure rather than the right timing. Adolescent cod liver oil users may be more likely to have additionally consumed the supplement during the other periods resulting in a longer exposure. However, when analyzing continuous supplementation from birth over increasingly longer periods, the strength of association did not steadily increase. The longest supplementation period was less strongly associated with a reduced MS risk than the shorter ones.

Lastly, our findings might be due to the protective effect of other cod liver oil ingredients. Vitamin A and E as well as omega-3 fatty acids have become of interest as possible disease-modifying candidates of MS.^31–33^ Fewer studies focused on a risk-modulating potential and results are not consistent. A U-shaped pattern of association between serum vitamin A levels and MS risk was reported in a registry-based smaller cohort study.^34^ However, intake of carotenoids, vitamin E and omega-3 fatty acids assessed by food frequency questionnaires was unrelated to disease risk in two large cohorts of women.^35,36^ We cannot exclude the possibility that vitamin A in cod liver oil contributed to our findings. The dose-response relationship observed for use during adolescence might be attributed to a protective effect of vitamin A, which increases along with vitamin D in higher doses of cod liver oil. It is unknown whether this nutrient acts age-dependently and could thus explain the findings on the timing of exposure to cod liver oil. Salzer and colleagues reported similar associations for younger participants (16–26 years) as in the entire cohort.^34^ Even if age at exposure was not addressed in detail, this observation contradicts somewhat the presence of a strong age-dependent effect for vitamin A. Moreover, evidence for an age-varying action of vitamin D exists beyond studies focusing on dietary sources, and thus independent of vitamin A.^4^

The MS prevalence is lower in populations with consumption of fish,^37^ and earlier studies suggested an inverse association between fish consumption and MS.^4,38^ Focusing on vitamin D-rich fatty fish, we found that consuming it during adolescence might be protective against the disease. The association was similar but no longer significant after adjusting for cod liver oil use. Our study might be underpowered to show subtle associations.

Previous studies investigating and comparing associations in different age periods in humans were smaller and could not account for all of the known risk factors of MS in the analyses.^4,5^ In this present large case-control study we analyzed cod liver oil supplementation, widely used in the Norwegian population, as a dietary proxy for vitamin D intake and serum levels. Investigating and comparing various age periods we found evidence of clear age differences in how vitamin D might affect MS risk even after adjusting for possible confounding.

Case-control studies, although efficient to conduct and suited to study past exposures in detail, are subject to methodological limitations. Despite the population-based sampling of both cases and controls, selection bias could, for instance, be an issue considering the different response rates between both groups. Compared to those included in the study, individuals not responding could have a different distribution of some of their main characteristics affecting the correlation between exposure and disease. We do not have any information on such a possible relation, but we found a higher proportion of controls with the highest level of education compared to the cases, indicating a higher socioeconomic status in controls. We accounted for this by adjusting for confounding by education.

Recall bias is another potential threat to the validity of findings in case-control studies. Nevertheless, the overall proportion of participants reporting cod liver oil use did not differ among cases and controls, and it is unlikely that participants were biased in recalling the timing of this supplementation. Knowledge about the period in life most susceptible to MS risk modification is not yet established.

In order to reduce the risk for misclassified responses, non-differential due to memory issues in general and differential due to deteriorating cognitive function in MS patients, only cases with a maximum disease duration of 10 years were eligible for the study. Furthermore, the age-period scale used to explore supplementation habits was adapted to the Norwegian school system to facilitate recall. In addition, participants were encouraged to ask their parents for help to correctly reconstruct past exposures if needed.

In conclusion, our findings suggest that adolescence might be an important postnatal age-period for an MS risk-reduction. Commonly used doses of vitamin D contained in cod liver oil might contribute to modify MS risk when supplemented throughout adolescence. Further studies are needed to confirm our findings and to investigate whether higher doses might potentially be as protective during childhood or adulthood.
